# Nuclei instance segmentation from histopathology images using Bayesian dropout based deep learning

**DOI:** 10.1186/s12880-023-01121-3

**Published:** 2023-10-19

**Authors:** Naga Raju Gudhe, Veli-Matti Kosma, Hamid Behravan, Arto Mannermaa

**Affiliations:** 1 Institute of Clinical Medicine, Pathology and Forensic Medicine, Multidisciplinary Cancer research community RC Cancer, University of Eastern Finland, P.O. Box 1627, Kuopio, 70211 Finland; 2 Biobank of Eastern Finland, Kuopio University Hospital, Kuopio, Finland

**Keywords:** Semantic segmentation, Bayesian deep learning, Uncertainty estimation, Nuclei segmentation, Digital pathology, Medical image analysis

## Abstract

**Background:**

The deterministic deep learning models have achieved state-of-the-art performance in various medical image analysis tasks, including nuclei segmentation from histopathology images. The deterministic models focus on improving the model prediction accuracy without assessing the confidence in the predictions.

**Methods:**

We propose a semantic segmentation model using Bayesian representation to segment nuclei from the histopathology images and to further quantify the epistemic uncertainty. We employ Bayesian approximation with Monte-Carlo (MC) dropout during the inference time to estimate the model’s prediction uncertainty.

**Results:**

We evaluate the performance of the proposed approach on the PanNuke dataset, which consists of 312 visual fields from 19 organ types. We compare the nuclei segmentation accuracy of our approach with that of a fully convolutional neural network, U-Net, SegNet, and the state-of-the-art Hover-net. We use F1-score and intersection over union (IoU) as the evaluation metrics. The proposed approach achieves a mean F1-score of 0.893 ± 0.008 and an IoU value of 0.868 ± 0.003 on the test set of the PanNuke dataset. These results outperform the Hover-net, which has a mean F1-score of 0.871 ± 0.010 and an IoU value of 0.840 ± 0.032.

**Conclusions:**

The proposed approach, which incorporates Bayesian representation and Monte-Carlo dropout, demonstrates superior performance in segmenting nuclei from histopathology images compared to existing models such as U-Net, SegNet, and Hover-net. By considering the epistemic uncertainty, our model provides a more reliable estimation of the prediction confidence. These findings highlight the potential of Bayesian deep learning for improving medical image analysis tasks and can contribute to the development of more accurate and reliable computer-aided diagnostic systems.

**Supplementary Information:**

The online version contains supplementary material available at 10.1186/s12880-023-01121-3.

## Introduction

The whole slide image (WSI) is the digital version of the patient-derived histology glass slide and provides ample opportunities to develop quantitative and qualitative profiling of the spatial patterns from the cancer tissues. The WSI contains hundreds of thousands of nuclei of various cell types, which is challenging to automatically segment based on the cell types. The manual assessment of the cell types from the hematoxylin and eosin (H &E) stained slides is prone to inter-and intra-observer variability [1]. Automating the workflow of the nuclei segmentation can accelerate the pathologist workflow in analyzing the nuclei cell morphology, cancer cell type classification, and grading [2]. The automatic nuclei segmentation allows computing the nuclei features, which can be used for predicting tissue-phenotype [3], tumor grading [4], estimating cancer recurrence rate [5, 6], and survival analysis [7]. Accurate segmentation of the nuclei from the H &E-stained histopathology images have several challenges due to the variations in the type of organ, tissue site, and variability between the sites, which produced the H &E-stained images [8].

Traditional computer vision algorithms, such as morphological image operations and watershed algorithms, are widely used for nuclei segmentation [8]. However, these algorithms are developed on limited set of images and often fail to generalize on new images. Recently, deep learning (DL) models, especially convolution neural networks, have achieved state-of-the-art performance in various medical image analysis tasks [9, 10]. Long et al. [11] proposed an encoder-decoder based fully convolutional neural network (FCN) for the semantic segmentation task. FCN consists of the contrasting path (encoder) with a set of convolutional layers to extract imaging features and the expanding path (decoder) with transpose or up-convolutions to reconstruct the extracted features and to segment the regions of interest in the input image. Inspired by [11], authors in [12] introduced skip connections to restore the spatial information lost during the contraction and expansion of the network and named the network, as U-Net. The classical U-Net model has been successfully incorporated into various medical image segmentation tasks. Despite being state-of-the-art model, U-Net often fails to segment overlapping and touching nuclei and requires post-processing techniques, such as watershed algorithm [13], for separating such nuclei. Several variants of the U-Net architecture have been proposed to improve image segmentation accuracy [14–17]. However, these studies focused on improving the accuracy and ignored uncertainty in the predictions. Graham et al. [2] introduced *Hover-net*, that can simultaneously perform the nuclei segmentation and classification. Hover-net incorporated horizontal and vertical distance maps to separate the touching nuclei, and demonstrated the state-of-the-art performance. Hover-net lacks the ability to quantify the uncertainty of nuclei segmentation and classification tasks.

The uncertainty quantification at pixel level is as crucial as model accuracy, especially among pathologist, to trust and incorporate DL algorithms in their medical diagnosis [18]. The uncertainty quantification explains the DL models’ overall confidence in predictions and improves the reliability in the decision-making process [18]. Typically, a DL model results in two kinds of uncertainties, *epistemic* and *aleatoric* [19]. The epistemic or model uncertainty often arises due to a lack of training data. Increasing the training data size often reduces the epistemic uncertainty. The aleatoric or data uncertainty usually arises due to the presence of noise in the data. This type of uncertainty cannot be reduced by increasing the data [19]. Bayesian methods provide a probabilistic representation of uncertainty and are widely-used for estimating the predictive uncertainties [18–23]. In addition to Bayesian methods, several other approximation methods, such as MC dropout [24], variational inference [25, 26], dropout variational inference [27], and ensemble learning [28], have been proposed for estimating uncertainty.

In this study, we present an encoder-decoder-based Bayesian DL model for nuclei instance segmentation from the H &E-stained histopathology images and estimate epistemic uncertainty by using the MC dropout approximation during the inference time. We demonstrate the efficiency of the proposed approach using a publicly available data from 19 different organs.

## Methodology

### Nuclei instance segmentation architecture

**Fig. 1:**
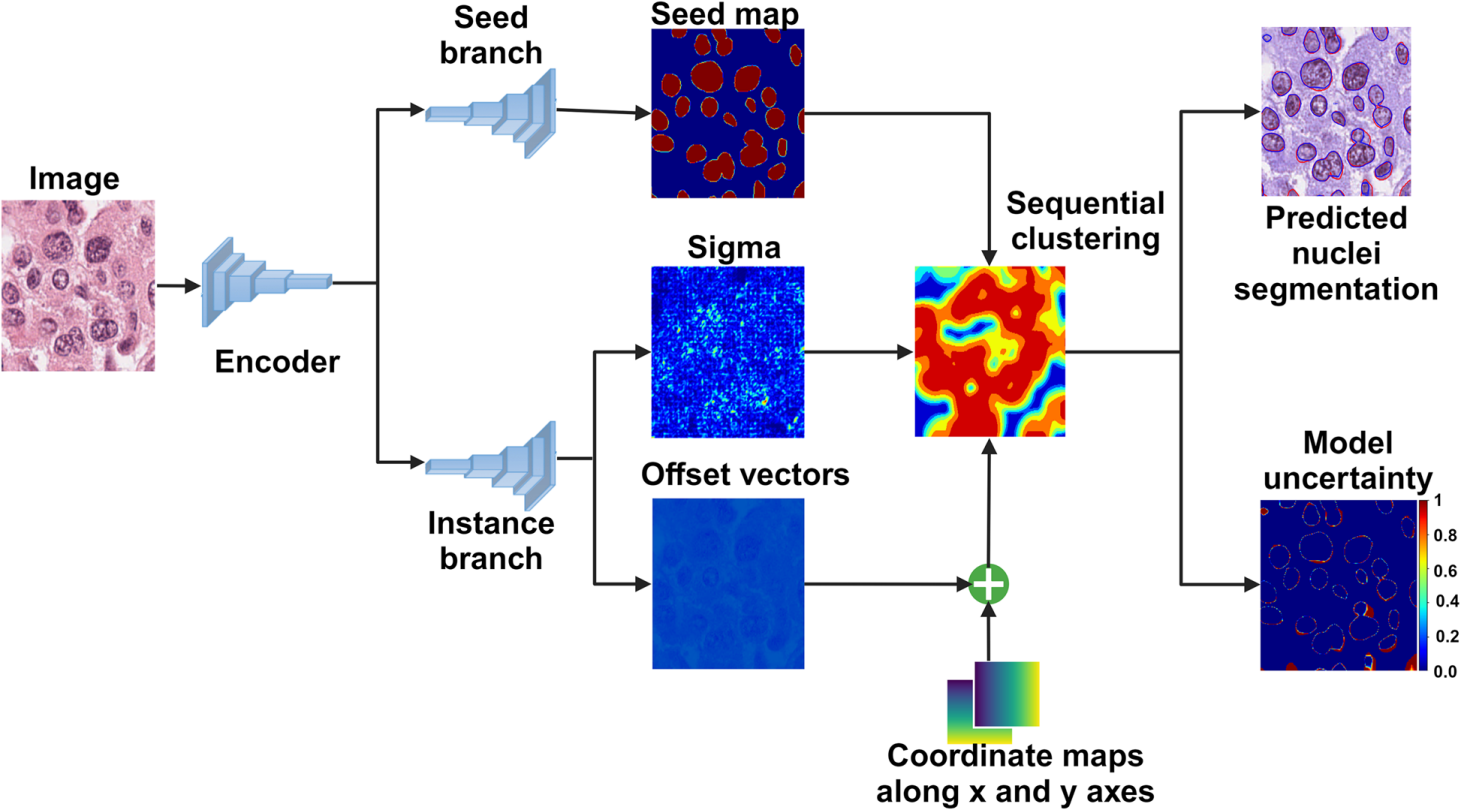
The BayesNuSeg model consists of one encoder and two independent decoders. The seed branch decoder predicts *k* seed maps for each class label. The instance branch decoder computes the offset vectors in both *x* and *y* dimensions, which are further added to the coordinate maps along the corresponding axes to obtain pixel embedding and mean of the instances (sigma). The seed maps, sigma, and pixels embedding are clustered using sequential clustering approach, which involves grouping similar pixels together based on their feature representation, to segment the nuclei by sampling the pixel embedding with the highest seed margin and using that coordinate location as instance center ${\textbf {C}}_k$. The output is the predicted nuclei segmentation and the model uncertainty quantification

**Fig. 2:**
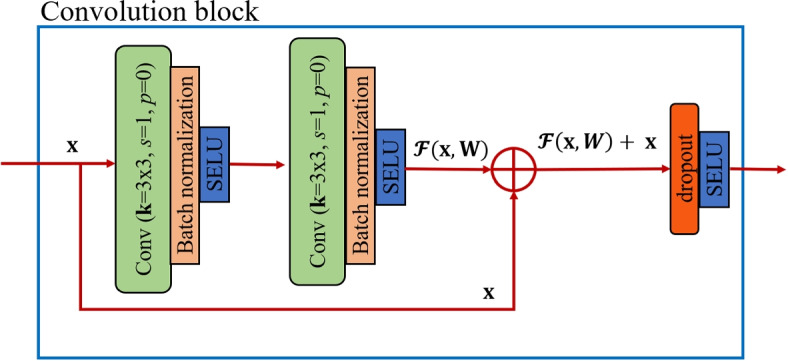
The convolution block of the BayesNuSeg model. The encoder unit contains five convolution blocks. The two independent decoder units contain the same number of convolution blocks, where a transpose convolution operation replaces the convolution operation. The parameter *k* represents the kernel size, *s* and *p* denote stride and padding, respectively. Finally, the dropout layer with a probability of 0.5 is activated during the inference phase to estimate the model uncertainty

We modified the network proposed by [29] into a Bayesian representation to simultaneously segment the nuclei and quantify the model uncertainty. The proposed model, named as *BayesNuSeg*, consists of an encoder and two independent decoders (Fig. 1). The encoder is a five-layered network, each containing a residual learning-based convolution followed by a batch-normalization [30] and a scaled exponential linear unit (SELU) [31], as shown in Fig. 2. The decoder replaces the convolution operation with a transpose convolution to reconstruct the extracted features. The seed branch decoder outputs the class specific seed maps and the instance branch decoder generates the pixel embedding.

The objective of the instance segmentation is to cluster a set of input image pixels ${\textbf {x}}\ =\ \{ x_1,\ x_2,\ x_3,\ \ldots ,x_N\ |\ x\ \in \ \mathcal {R}^2\}$ into a set of instances ${\textbf {s}}\ =\ \{ s_1,\ s_2,\ \ldots ...,s_k\}$. The discriminative learning function $\mathcal {F}({\textbf {x}},{\textbf {W}})$, is employed to localize different nuclei in H &E-stained histology images **x**, utilizing the weight parameter matrix **W**, in order to accomplish the instance segmentation task. The instance branch of the decoder network, maps each pixel $x_i$ of the given input image **x**, to an offset vector ${\textbf {o}}_{\textbf {i}} \in \mathcal {R}^2$ from which the pixel embeddings, ${\textbf {e}}_{\textbf {i}}= x_i + {\textbf {o}}_{\textbf {i}},$ are generated and pointing to their corresponding instance centroid, ${\textbf {C}}_k\ =\ \frac{1}{N}\ \sum _{x\ \in \ S_k} x$. The size and shape of the nuclei vary within each cell type, therefore, to ensure pixels of one instance are close to their centroid, an instance specific margin loss function is used [29]. A Gaussian function $\phi _k$ for each instance $s_k$ converts the distance between ${\textbf {e}}_i$ and ${\textbf {C}}_k$ into a probability of belonging to an instance $s_k$:

1 $$\begin{aligned} \phi _k({\textbf {e}}_i) = \exp \left( -\frac{({\textbf {e}}_{kx} - {\textbf {C}}_{kx})^2}{2 \times \sigma _{kx}^2} -\frac{({\textbf {e}}_{ky} - {\textbf {C}}_{ky})^2}{2 \times \sigma _{ky}^2} \right) . \end{aligned}$$

In addition to the offset vectors, the instance decoder branch computes the standard deviation (sigma), ${\sigma }k \in R^2$, for each instance $s_k$. The value of ${\sigma }k$ indicates the proximity of the pixel embedding $\textbf{e}_i$ to the instance centroid $\textbf{C}_k$: a higher ${\sigma }_k$ suggests the pixel ${\textbf {e}}_{{\textbf {i}}}$ is likely part of instance $s_k$, whereas a lower value suggests it belongs to the background.

To classify a pixel ${\textbf {e}}_i$, a threshold of $\phi _k\left( {\textbf {e}}_i\right) \ge 0.5$ is applied. This threshold represents the decision boundary at which the probability of a pixel belonging to an instance $s_k$ or the background is equal. Specifically, a pixel ${\textbf {e}}_i$ is assigned to instance $s_k$, if $\phi _k\left( {\textbf {e}}_i\right) \ge 0.5$, indicating a higher probability of belonging to instance $s_k$ than to the background, and vice versa.

The seed decoder branch computes the seediness score, the likelihood that the pixel $x_i$ belongs to the instance $s_k$. Sequential clustering is employed on the aggregated offset vectors, sigma and seediness score to group the pixels that belong to the same instance, and finally provides the segmented nuclei mask. To train the model end-to-end, the combined loss function contains three terms as follows [29]:

2 $$\begin{aligned} {\textbf {L}} = \lambda _{\text {IoU}} \times {\textbf {L}}_{\text {IoU}} + \lambda _{\text {seed}} \times {\textbf {L}}_{\text {seed}} + \lambda _{\text {smooth}}\times {\textbf {L}}_{\text {smooth}}, \end{aligned}$$

where, $\lambda _{\text {IoU}}$, $\lambda _{\text {seed}}$ and $\lambda _{\text {smooth}}$ are the hyper-parameters of the combined loss function. We used $\lambda _{\text {IoU}}=1$, $\lambda _{\text {seed}}=1$, and $\lambda _{\text {smooth}}=10$ , as suggested by [29]. Additional details for the combined loss function **L** are provided in Appendix A.

### Bayesian uncertainty representation

We follow the [27] uncertainty estimation approach by applying the dropout technique, as the variational approximation (see Appendix B for the overview of Bayesian representation learning). To quantify the model uncertainty, we use MC dropout to approximate the predictive variance at the inference time, as follows [19]:

3 $$\begin{aligned} {\hat{\sum }}^2\ =\ \frac{1}{T}\ \sum _{t=1}^{T}{(\widehat{\text {y}}_t\ \ -\ \frac{1}{T}\sum _{t=1}^{T}\widehat{\text {y}}_t\ )^2\ +\ \frac{1}{T}\ \sum _{t=1}^{T}{\widehat{\sigma }_t}^2}, \end{aligned}$$

where, ${\hat{\sum }}^2$ is the measure of the model uncertainty, $\sigma _t$ is the standard deviation of the predicted segmentation mask, $\hat{\text {y}}_t$, and *T* represents the number of stochastic forward passes of MC dropout sampling. The mathematical derivation of Eq. 3 is given in Appendix C. We used $T = 50$ in our experiments, as the optimal number of MC dropout sampling. The effect of changing *T* on the model performance in terms of F1-score is presented in Appendix D.

## System set-ups

### Dataset

**Fig. 3:**
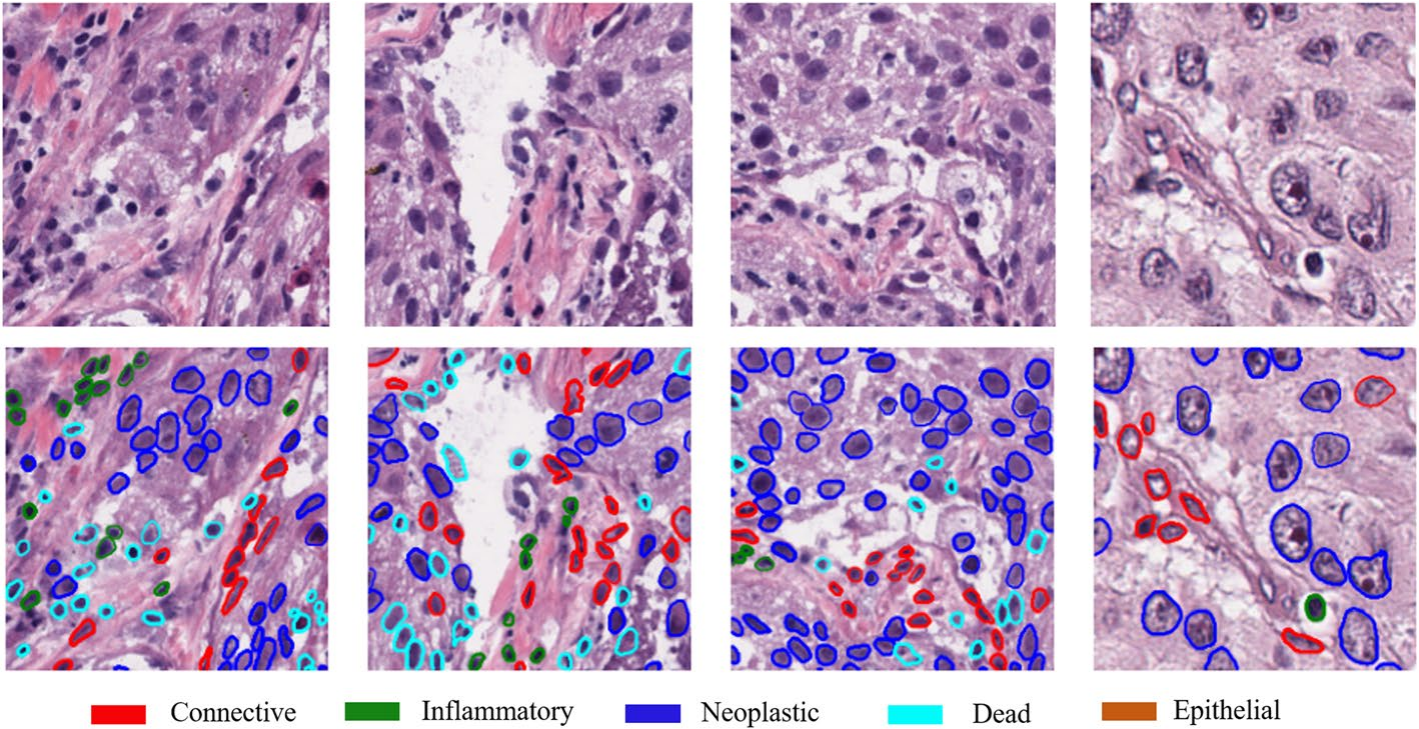
Examples of images and ground truth masks for nuclei segmentation from the PanNuke dataset. The first row represents the H &E-stained images and the second row represents the ground truth binary masks. The different color contours represent different nucleus types, as shown in the legends

We trained and validated the BayesNuSeg and the other baseline approaches using PanNuke dataset [32]. The dataset has variability in the image staining protocol and has been collected from different sites. The dataset contains 312 visual fields from 19 different organs randomly sampled at different resolutions from more than twenty thousand WSIs of the Cancer Genome Atlas (TCGA) [33]. The dataset organizers provided the data in 3 folds with a total of 7901 images with their corresponding ground truth masks at a resolution of $256\times 256$. A visual example of the images and ground truth masks are illustrated in Fig. 3.

### Implementation details

**Fig. 4:**
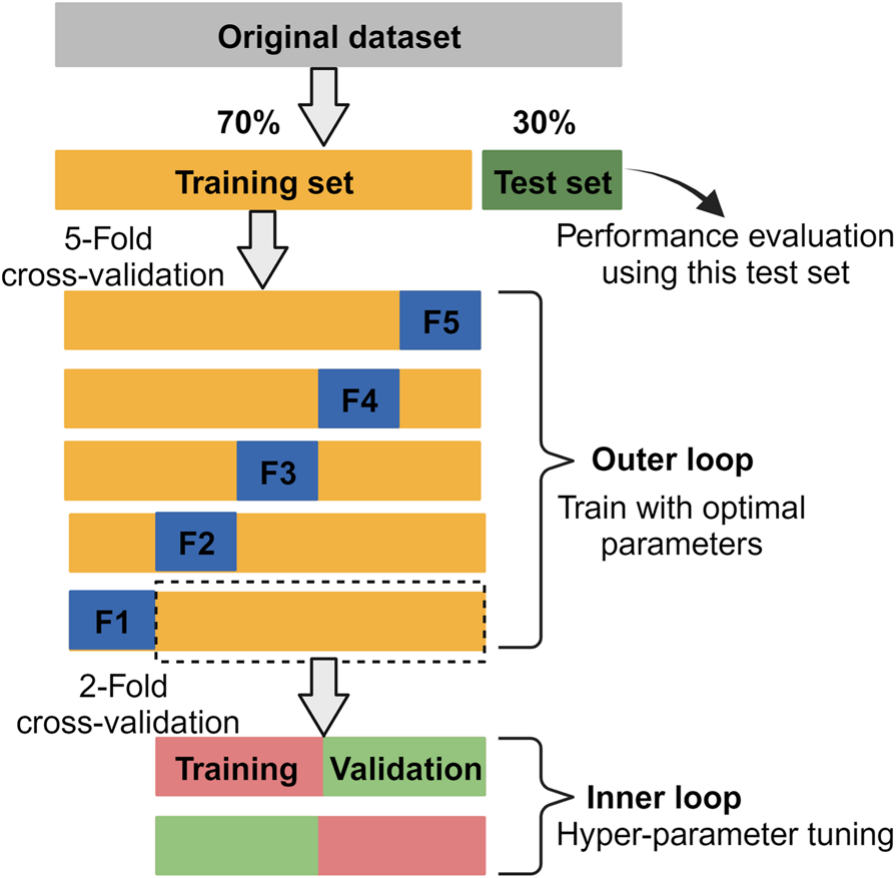
Illustration of the nested cross-validation used in this study. Figure adopted from [36] and re-created using bio-render (https://app.biorender.com/)

Data variability, due to differing H &E-staining protocols across various organs, was mitigated by applying the Vahadane stain normalization technique [34], utilizing a reference image from the stomach organ. We implemented nested cross-validation strategy to train, tune and evaluate the proposed and baseline models. The dataset was first divided into two distinct subsets: 70% as model development set (encompassing training, hyper-parameter tuning and validation, 5530 images), and the remaining 30% reserved as an external test set (2371 images), as depicted in Fig. 4. The model development set was subjected to nested cross-validation, involving an outer k-fold loop for model training and evaluation, and an inner loop for hyper-parameter tuning using Optuna [35]. Within each fold of the outer loop, the model was trained on a fraction of $\frac{(k-1)}{k}$ of the data, while hyper-parameters were optimized using an inner cross-validation loop on this subset. The model was then evaluated on the remaining $\frac{1}{k}$ of the data. This methodological design ensure optimal hyper-parameter tuning for each outer loop fold, yielding a robust and unbiased model performance. Following the optimal hyper-parameters and subsequent model training with $k=5$ folds, performance was evaluated on the external test set to obtain an unbiased evaluation of the model’s ability to generalize to unseen data.

To minimize over fitting, several augmentation techniques [37] were also employed during training, including horizontal and vertical flips, random rotations, and random color jitters. The training was executed on a NVIDIA Tesla V100 GPU, provided by the CSC-IT Center for Science, Finland [38]. During the inference phase, the MC-dropout technique was employed to estimate model uncertainty, resulting in the BayesNuSeg model (with uncertainty).

### Evaluation metrics

We evaluated the performance of the proposed BayesNuSeg and the baseline models, FCN, U-Net, SegNet, and Hover-net using F1-score and IoU, as the evaluation metrics. F1-score is the harmonic mean between precision and recall. A higher F1-score indicates a better intersection between the ground truth and the predicted segmentation mask. The IoU, also referred as the Jaccard index, is used to quantify the percentage of overlap between the ground truth and the predicted segmentation mask [39].

Further, during the inference time, we applied MC dropout for BayesNuSeg and all the baseline models and computed the uncertainty accuracy (UA) defined by [40]. A higher UA value indicates a higher level of confidence in the model’s predictions. To report our results, we used a 95% confidence interval as a measure of dispersion [41] for all the metrics.

## Results

### BayesNuSeg outperforms the baseline models in nuclei segmentation with enhanced accuracy and reliability

Table 1 reports nuclei segmentation results on the test set of the PanNuke dataset. BayesNuSeg with uncertainty outperforms all the baseline models. In particular, the proposed model achieves an F1-score of 0.893 ± 0.008, which outperforms state-of-the-art Hover-Net with F1-score of 0.871 ± 0.010, that is a relative improvement of 2.53%. Additionally, BayesNuSeg demonstrates its ability to estimate uncertainty accurately, as reflected by its UA score of 0.796± 0.001. The relative improvements of 6.31% and 5.43% over U-Net and FCN8, respectively, in terms of F1-score, further emphasize the superiority of BayesNuSeg. These findings show the potential of BayesNuSeg, with its uncertainty estimation capability, to enhance nuclei segmentation accuracy and reliability.

**Table 1:** The nuclei segmentation results of the BayesNuSeg and the baseline models. The BayesNuSeg model with uncertainty estimation outperforms all the baseline systems. N.A.: Not available

Method	F1-score	IoU	UA
**FCN8**	0.842 ± 0.008	0.732 ± 0.049	N.A.
**U-Net**	0.824 ± 0.009	0.791 ± 0.048	N.A.
**SegNet**	0.845 ± 0.018	0.803 ± 0.055	N.A.
**Hover-net**	0.851 ± 0.010	0.829 ± 0.032	N.A.
**BayesNuSeg**	0.848 ± 0.013	0.835 ± 0.003	N.A.
**FCN8 + MC dropout**	0.848 ± 0.009	0.764 ± 0.004	0.699 ± 0.050
**U-Net + MC dropout**	0.840 ± 0.009	0.804 ± 0.037	0.738 ± 0.034
**SegNet + MC dropout**	0.847 ± 0.006	0.828 ± 0.045	0.763 ± 0.046
**Hover-net + MC dropout**	0.871 ± 0.010	0.840 ± 0.031	0.789 ± 0.032
**BayesNuSeg + MC dropout**	**0.893** $\varvec{\pm }$ **0.008**	**0.868** $\varvec{\pm }$ **0.003**	**0.796** $\varvec{\pm }$ **0.004**

Bold font are the best values

**Fig. 5:**
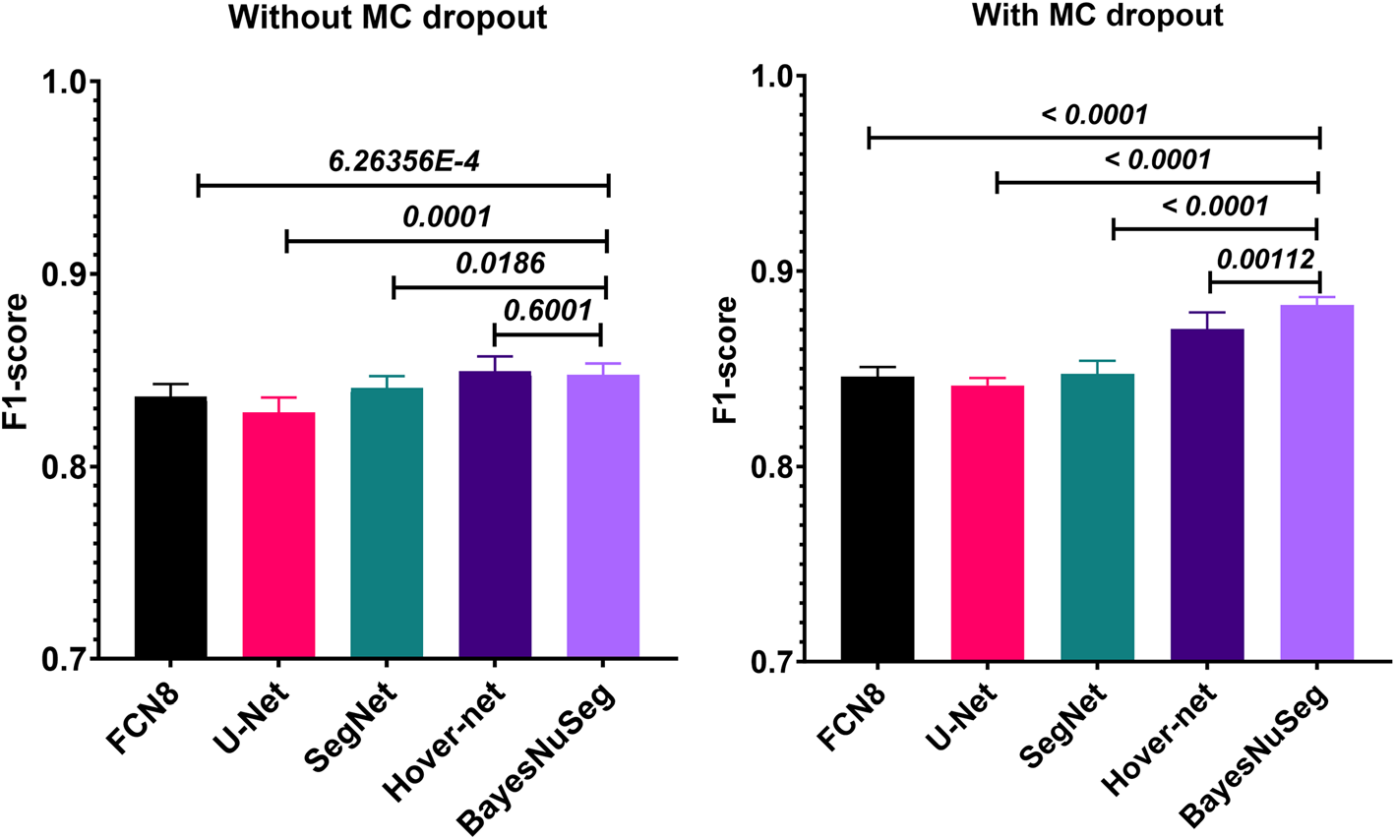
Statistical significance for the difference in F1-score of the proposed and the baseline approaches using two sample *t*-test with Welch correction. The difference is statistically significant if the *p*-value $\le 0.05$. The Y-axis depicts the overall F1-score achieved on the test set. The figure created using the GraphPad Prism software (https://www.graphpad.com/)

Additionally, we conducted two sample *t*-tests using the Welch correction to assess the statistical significance of the BayesNuseg model compared to various baseline models. The results, presented in Fig 5, indicate that the proposed approach is statistically significant when compared to most baseline models (with a *p*-value $\le 0.05$). The only exception is the Hover-net model without MC-dropout at inference time (*p*-value = 0.6001).

**Fig. 6:**
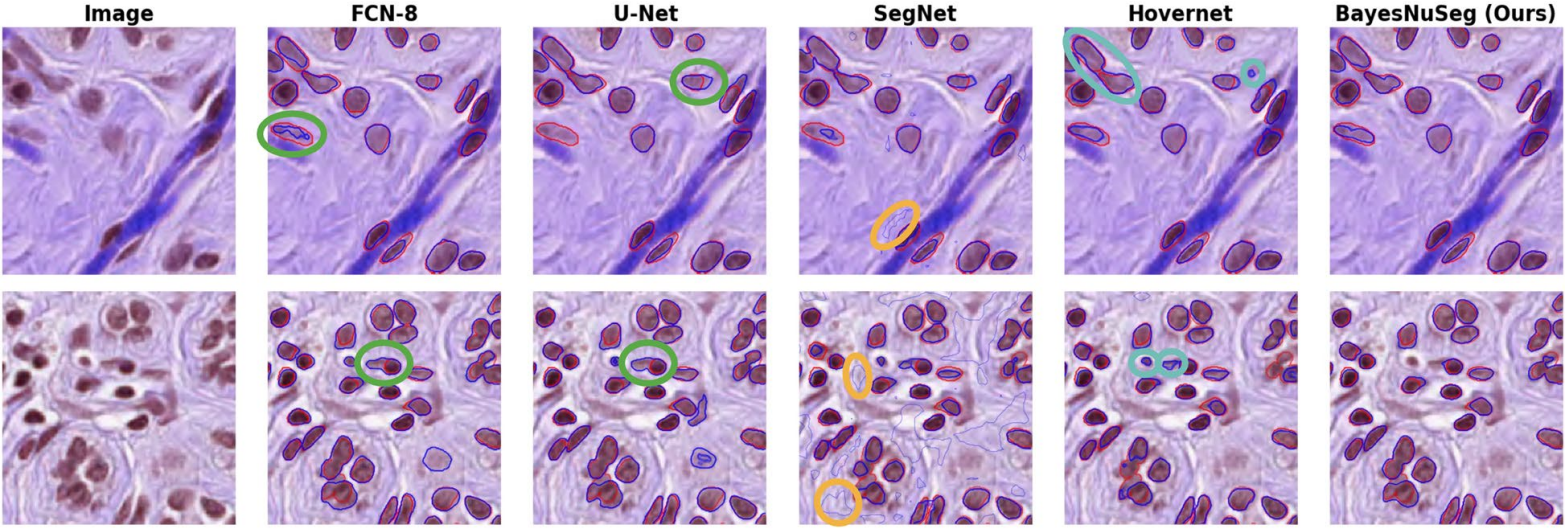
Visual assessment of the nuclei segmentation of the BayesNuSeg with uncertainty and the baseline models. For the visualization purposes, we combined all the nuclei cell types and showed the nuclei boundaries as a contour. The first column is the original images, and the next columns represent the predicted nuclei segmentation overlaid on the original image by the models. The red contours represent the ground truth annotations provided by the expert pathologist of the PanNuke dataset, whereas the blue contours indicate the nuclei segmentation as predicted by the proposed and other baseline approaches. We annotated the predictions of the baseline approaches using green, orange, and cyan circles (with thick contours). The green circle indicates that FCN8 and U-Net have failed to accurately estimate the nuclei boundaries. The orange circle highlights the noise present throughout the image due to the predictions of the SegNet approach. The cyan circle indicates the overestimation of predicted nuclei boundaries by the Hovernet approach

The qualitative results of the BayesNuSeg with uncertainty and other baseline models are illustrated in Fig. 6. The BayesNuSeg delineates the nuclei more preciously than the baseline approaches. The FCN and U-Net models identify all the nuclei in the image but fails to estimate the nuclei boundaries. As the number of nuclei cells increases, the FCN and U-Net models do not properly localize the nuclei having obscure boundaries, as shown with the green circles in Fig. 6. SegNet predictions contain noise in the segmentation, as shown with the orange circles in Fig. 6. Notice that the noise is distributed across the entire image in the first and second rows, fourth column. For the sake of simplicity, we have highlighted only a few areas. We additionally trained the SegNet model for 500 epochs. This reduced the noise in the segmentation; however, the accuracy was not significantly improved. We also noticed that the Hover-net sometimes fails to separate the touching nuclei and thus over-estimates the nuclei, as shown in the cyan color circles in Fig. 6. The BayesNuSeg model separates the touching nuclei more efficiently than the Hover-net. We suggest that the efficient estimation of pixel embeddings of each nuclei by the instance branch and localization of the nuclei by the coordinate maps are likely to contributes towards this success. The qualitative visualizations on few more examples are illustrated in the Appendix E.

### Applying MC dropout during inference reveals the robust uncertainty quantification abilities of the BayesNuSeg model

**Fig. 7:**
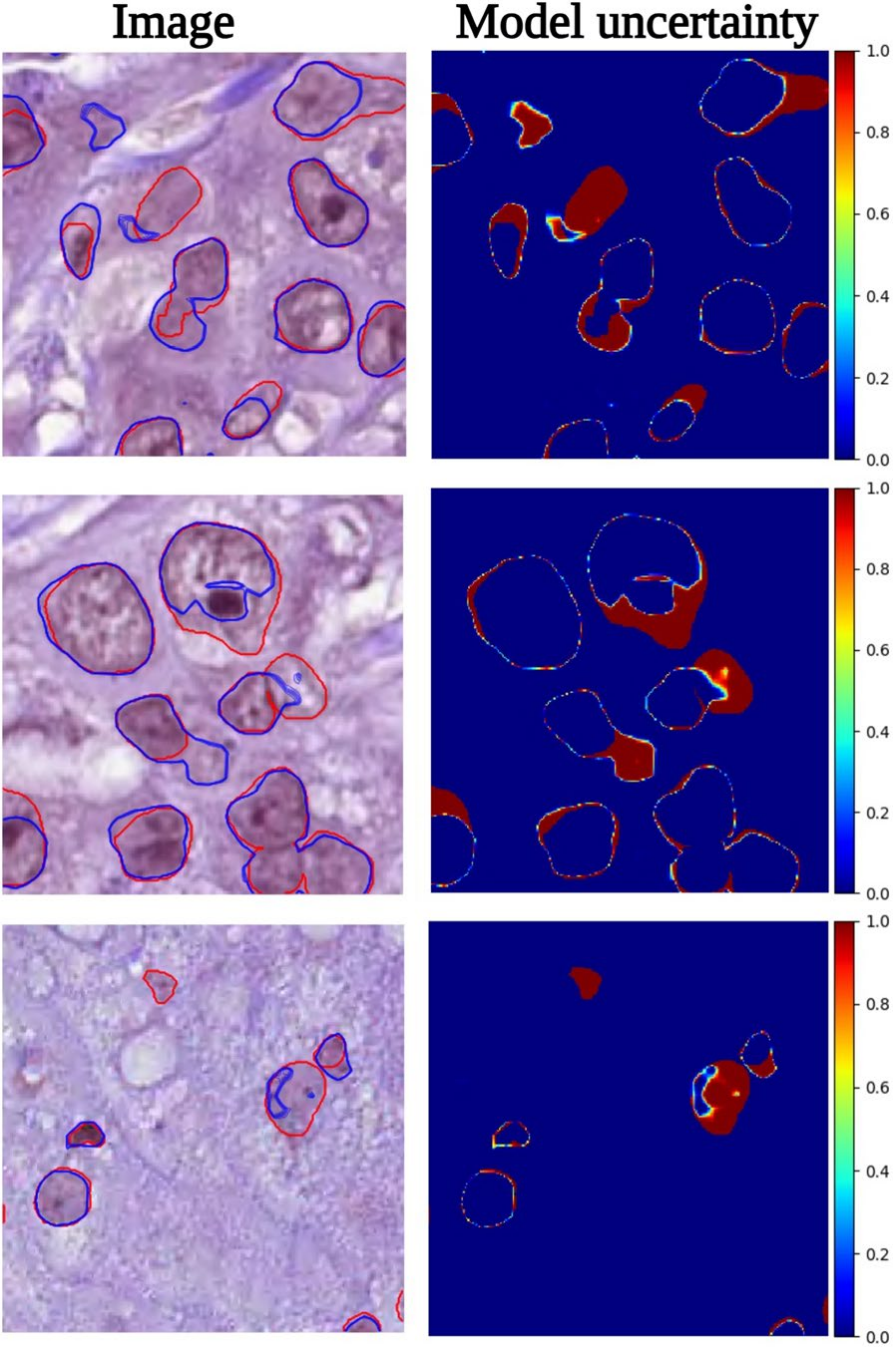
Uncertainty quantification estimated by the BayesNuSeg on the test set of the PanNuke dataset. The first column contains the ground truth nuclei (red contours) and the mean of MC dropout sampling predictions (blue contours), overlaid on the original image. The color bar next to the model uncertainty represents the uncertainty in range [0, 1], where 0 denotes lower and 1 highly uncertain predictions

Here, we demonstrate the uncertainty quantification using MC dropout approximation for $T=50$ samplings of the posterior distributions of the BayesNuSeg predictions. In Fig. 7, each row presents the uncertainty visualization of the given image. The model uncertainty was measured in range [0, 1], where 0 represents low uncertainty and 1 highly uncertain prediction. As shown, higher uncertainty is observed with the nuclei pixel intensities close to the background pixels, where the BayesNuSeg failed to identify or miss detected the nuclei. See Appendix F for few more examples.

## Discussion

The proposed BayesNuSeg model holds immense potential for advancing the field of digital pathology and has profound implications for both clinical practice and biological research. Pathologists and experienced researchers in the medical and AI domains can greatly benefit from the capabilities offered by this model.

One of the critical aspects of digital pathology is the quantification of nuclear features, such as size, shape, and texture, which play vital roles in understanding cellular morphology and tissue composition. The BayesNuSeg model provides precise segmentation results with an F1-score of 0.893 ± 0.008, enabling the extraction of nuclei features. Accurate nuclei segmentation can be utilized for quantitative analysis and characterization of tissue structures, ultimately aiding in the diagnosis, grading, and prognosis of disease. Precise nuclei segmentation serves as a foundational step for subsequent analyses, including cell counting, spatial arrangement analysis, and nuclei clustering. These analyses provide insights into cellular interactions, tissue organization, and pathological alterations at the cellular level. By unraveling disease mechanisms, identifying novel biomarkers, and advancing our understanding of complex biological processes, this model contributes to the forefront of biomedical research. Although, Hover-net segments nuclei precisely, the model demands additional post-processing techniques such as horizontal and vertical maps to separate the touching nuclei. The sequential clustering adopted in BayesNuseg, assists in identifying the pixels belonging to similar instances and thus avoid for additional post-processing steps.

BayesNuSeg’s robust uncertainty quantification capability enables the identification of challenging regions where the model may struggle to accurately segment nuclei. This insight assists clinicians focus their attention on areas requiring additional scrutiny, thereby enhancing diagnostic accuracy and reducing the potential for misinterpretations.

It is important to acknowledge the limitations of our study. We solely evaluated the BayesNuSeg model using the PanNuke dataset, which consists of whole-slide images (WSIs) from various organs. While this dataset encompasses variability in image staining protocols representative of challenges encountered in other datasets, a more comprehensive evaluation on a broader range of datasets would be beneficial. This would allow for a more robust assessment of the model’s performance. Nonetheless, our results clearly demonstrate that BayesNuSeg outperformed several established and state-of-the-art models in terms of WSI segmentation on the PanNuke dataset.

## Conclusion

We presented the Bayesian dropout based deep learning representation for nuclei segmentation from H &E-stained medical images. We showed the performance of our proposed BayesNuSeg model for nuclei segmentation and uncertainty qualification on the PanNuke dataset containing 312 pathology slides from 19 different organs. We selected the FCN, the U-Net, the SegNet and the Hover-Net, as the baseline models for comparison. The proposed model with uncertainty achieves an F1-score of 0.893 ± 0.008 which outperforms the state-of-the-art Hover-net with F1-score of 0.871 ± 0.010, that is a relative improvement of 2.53%. Additionally, we validated the efficacy of our proposed model by leveraging MC dropout sampling as an approximation of the posterior distribution for uncertainty quantification. In our next study, we will use the output of the BayesNuSeg model to study tumor micro-environment and to identify the breast cancer tumor biomarkers from the H &E-stained pathology images.

### Supplementary Information

**Additional file 1:** **Appendix A.** End-to-end trainable loss function. **Appendix B.** Overview of the Bayesian approximation. **Appendix C.** Measure of predictive variance. **Appendix D.** MC dropout sampling optimization experiment. **Appendix E.** Visualization of the segmentation outputs by the BayesNuSeg and the baseline models. **Appendix F.** Model’s uncertainty quantification using BayesNuSeg model.

## Data Availability

The PanNuke imaging dataset and the corresponding annotation used in this study are publicly available data. The dataset has been made open source and is available for download from https://warwick.ac.uk/fac/cross_fac/tia/data/pannuke. The working codes are available at https://github.com/uefcancer/BayesNuSeg.

## Abbreviations

MC: Monte-Carlo
IoU: Intersection over union
WSI: Whole slide image
H[MYAMP: E] Hematoxylin and eosin
DL: deep learning
FCN: Fully convolutional neural network
Encoder: Contrasting path
Decoder: Expanding path
SELU: Scaled exponential linear unit
sigma: Standard deviation
TCGA: Cancer Genome Atlas
UA: Uncertainty accuracy
