# Supplementary material for: Nuclei instance segmentation from histopathology images using Bayesian dropout based deep learning
Source: BMC Med Imaging. 2023 Oct 19;23:162. doi: 10.1186/s12880-023-01121-3 (PMC10585914; doi:10.1186/s12880-023-01121-3)
Supplement: Supplementary file 1 — Additional file 1: Appendix A. End-to-end trainable loss function. Appendix B. Overview of the Bayesian approximation. Appendix C. Measure of predictive variance. Appendix D. MC dropout sampling optimization experiment. Appendix E. Visualization of the segmentation outputs by the BayesNuSeg and the baseline models. Appendix F. Model’s uncertainty quantification using BayesNuSeg model. [file 12880_2023_1121_MOESM1_ESM.pdf]

Nuclei instance segmentation from histopathology images using Bayesian dropout based deep learning

Supplementary information.

Appendix A End-to-end trainable loss function

To train the BayesNuSeg model end-to-end, the combined loss function of the two decoders consists of three loss functions $\mathbf{L}_{\text{IoU}}$, $\mathbf{L}_{\text{seed}}$, and $\mathbf{L}_{\text{smooth}}$ as shown in Eq. 2 (main article) $\mathbf{L}_{\text{IoU}}$ is Lovasz-SoftMax loss [1] and is computed by using the predicted ϕ_k of each image pixel and the ground truth instance mask s_k. Minimizing this loss directly optimizes the mean IoU between the predicted and the ground-truth segmentation mask. $\mathbf{L}_{\text{IoU}}$ loss cannot alone differentiate the pixels belonging to the background [2]. To resolve this issue, $\mathbf{L}_{\text{seed}}$ computes the seediness value of each instance s_k, such that the seediness values tend to zero if the image pixel belongs to the background and to $\phi_k(\mathbf{e}_i)$, if the pixels belong to the foreground. The seed loss L_{seed} is computed by [2]:

$$\mathbf{L}_{\text{seed}} = \frac{1}{N} \sum_{i=1}^{N} \lambda_{fg} \mathbf{I}_{\{s_i \in s_k\}} \left\| s_i - \phi_k(\mathbf{e}_i) \right\|^2 + \lambda_{bg} \mathbf{I}_{\{s_i \in s_{bg}\}} \left\| s_i - 0 \right\|^2, \tag{A1}$$

where, s_i is the network's seed output of pixel x_i, and λ_{fg} and λ_{bg} are the hyper-parameters of foreground and background image pixels, respectively. We set λ_{fg} and λ_{bg} as 10 and 1, respectively, as suggested by [2]. The pixels in the seed map with the highest score indicate that the embeddings are close to object center. This procedure is repeated until all the seed maps are masked. An additional smoothness term is added to the loss function to ensure that the $\hat{\sigma}_k \approx \sigma_k = \frac{1}{|s_K|} \sum_{\sigma_i \in S_K} \sigma_i$. The smoothness loss function $\mathbf{L}_{\text{smooth}}$ is computed by [2]:

$$\mathbf{L}_{\text{smooth}} = \frac{1}{|S_K|} \sum_{\sigma_i \in s_k} \left\| \sigma_i - \sigma_k \right\|^2. \tag{A2}$$

Appendix B Overview of the Bayesian approximation

Given a dataset of images and their corresponding segmentation masks $\mathcal{D} = \{(x_1, y_1), (x_2, y_2), \ldots, (x_N, y_N)\}$, using the Bayes theorem, the posterior distribution over the weight parameters $\mathbf{W}$ is defined as, $p(\mathbf{W}|x, y) = \frac{p(y|x,\mathbf{W})p(\mathbf{W})}{p(y|x)}$, where $p(y|x, \mathbf{W})$ is the likelihood function and $p(y|x)$ is the marginal likelihood.

Let us assume that y^* is the predicted output segmentation of an unseen image x^*, the deterministic learning function is represented as $p(y^* = c|x^*) = Softmax(\mathcal{F}(x^*, \mathbf{W}))$, for c categorical class labels. The probabilistic Bayesian network is represented as:

$$p(y^* = c|x^*) = \int p(y^* = c|x^*, \mathbf{W})p(\mathbf{W} \mid x, y)d(\mathbf{W}). \tag{B3}$$

It is hard to approximate the posterior distribution using Eq. B3. [3] proved that approximation of the posterior distribution is equivalent to the dropout, a regularization technique in neural networks with a Bernoulli distribution $q(\hat{\mathbf{W}})$, where $\hat{\mathbf{W}} = \mathbf{W} \,.\, diag(z)$, $z \sim Bernoulli\,(\theta)$ with θ as the dropout value, and '.' indicates the dot product. Posterior distribution in Eq. B3 can be approximated by minimizing the Kullback-Leibler (KL) divergence between $q(\hat{\mathbf{W}})$ and $p(\hat{\mathbf{W}}x,\ y)$, as follows [3]:

$$p\left(y^* = c|x^*, x,\ y\right) = \int p\left(y^* = c \middle| x^*, \hat{\mathbf{W}}\right) q\left(\hat{\mathbf{W}}\right) d\hat{\mathbf{W}}. \tag{B4}$$

Appendix C Measure of predictive variance

Given T MC dropout samples, $\mathcal{F}(x, \mathbf{W}_t) = [\widehat{y}_t, \widehat{\sigma}_t^2]$ from the approximate predictive distribution $p(y^*|\ x^*,\ x,\ y\ ) = \int p\left(y^* \middle| x^*, \hat{\mathbf{W}}\right) q\left(\hat{\mathbf{W}}\right) d\hat{\mathbf{W}}$, the predictive sample variance $\hat{\sum}^2 = \frac{1}{T} \sum_{t=1}^{T} (\widehat{y}_t - \frac{1}{T} \sum_{t=1}^{T} \widehat{y}_t\ )^2 + \frac{1}{T} \sum_{t=1}^{T} \widehat{\sigma}_t^2$ is an unbiased estimator of the approximate predictive variance, i.e., the variance among the predictions can be used as an estimate of epistemic uncertainty.

Proof:

$$\mathrm{E}\left[\hat{\sum}^2\right] = \mathbb{E}\left[\frac{1}{T} \sum_{t=1}^{T} (\widehat{y}_t - \frac{1}{T} \sum_{t=1}^{T} \widehat{y}_t\ )^2 + \frac{1}{T} \sum_{t=1}^{T} \widehat{\sigma}_t{}^2\right]$$

$$= \mathrm{E}\left[\frac{1}{T} \sum_{t=1}^{T} (\widehat{y}_t - \frac{1}{T} \sum_{t=1}^{T} \widehat{y}_t\ )^2\right] + \mathbb{E}\left[\frac{1}{T} \sum_{t=1}^{T} \widehat{\sigma}_t{}^2\right].$$

With $\bar{y} = \frac{1}{T} \sum_{t=1}^{T} \widehat{y}_t$ follows

$$= \mathrm{E}\left[\frac{1}{T} \sum_{t=1}^{T} (\widehat{y}_t - \bar{y}\ )^2\right] + \hat{\sigma}^2$$

$$= \mathrm{E}\left[\frac{1}{T} \sum_{t=1}^{T} (\widehat{y}_t - y)^2 - (\ \bar{y} - y)^2\ \right] + \hat{\sigma}^2$$

$$= \mathrm{E}\big[(\ \hat{\mathrm{y}}\ \ -\ \mathrm{y})^2\big]\ -\ \mathbb{E}\left[(\ \bar{\mathrm{y}}\ -\ \mathrm{y})^2\right]\ \ +\ \hat{\sigma}^2$$

$$= \sum^2 - \hat{\sigma}^2\ +\ \hat{\sigma}^2$$

$$\mathbb{E}\left[\hat{\sum}^2\right] = \sum^2 .$$

Appendix D MC dropout sampling optimization experiment

Figure D1 demonstrates the effect of changing T, the number of MC dropout samplings. We computed the average mean of T sampled predicted segmentation of the BayesNuSeg model and evaluated the performance using F1-score and the validation set.

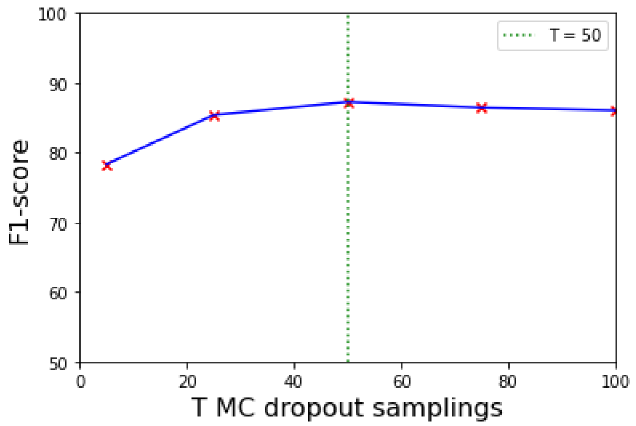

Fig. D1 Effect of changing T, the MC dropout samplings, to estimate the mean BayesNuSeg predicted segmentation. $T = 50$ achieves the best F1-score.

Appendix E Visualization of the segmentation outputs by the BayesNuSeg and the baseline models.

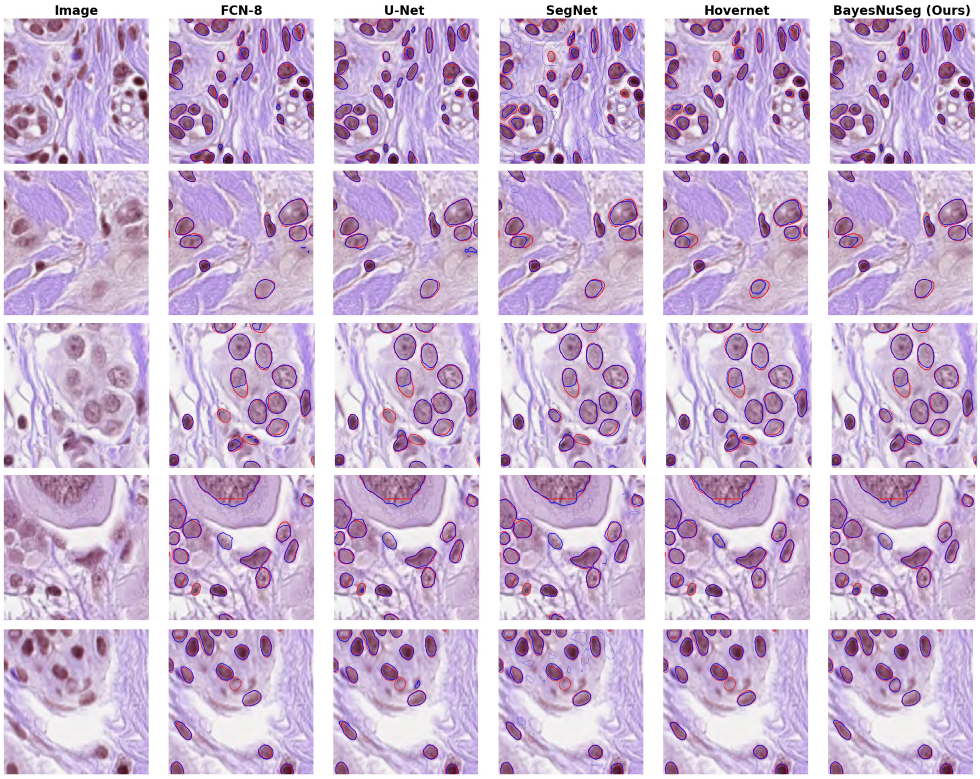

Fig. E2 Visual assessment of the nuclei segmentation of the BayesNuSeg with uncertainty and the baseline models. The BayesNuSeg better localizes the nuclei and separates the touching nuclei. The red and the blue contours denote the ground truth and the predicted nuclei segmentation by the models, respectively.

Appendix F Model's uncertainty quantification using BayesNuSeg model

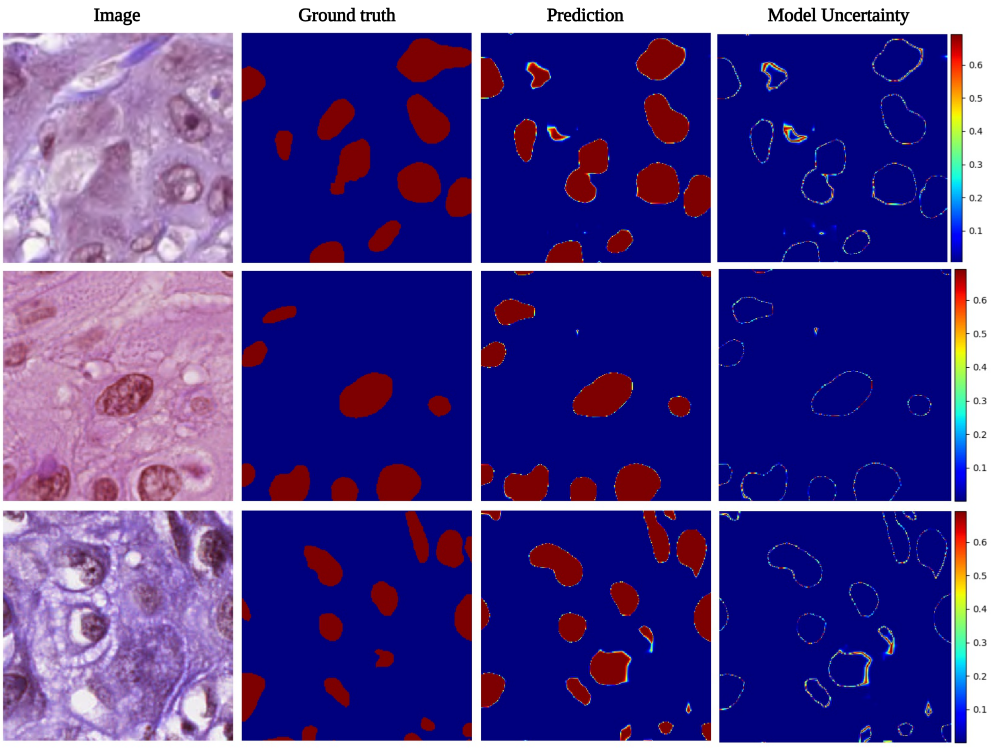

Fig. F3 Quantitative visualizations of uncertainty quantification using BayesNuSeg model. The first column represents the input image, the second and the third rows represent the ground truth and the predicted nuclei segmentation, respectively. The last column represents the uncertainty quantification in range [0, 1], with 1 indicating as the highest uncertainty.

References

[1] Berman, M., Triki, A.R., Blaschko, M.B.: The lovász-softmax loss: A tractable surrogate for the optimization of the intersection-over-union measure in neural networks. In: Proceedings of the IEEE Conference on Computer Vision and Pattern Recognition, pp. 4413–4421 (2018)

[2] Neven, D., Brabandere, B.D., Proesmans, M., Gool, L.V.: Instance segmentation by jointly optimizing spatial embeddings and clustering bandwidth. In: Proceedings of the IEEE/CVF Conference on Computer Vision and Pattern Recognition,

pp. 8837–8845 (2019)

[3] Gal, Y., Ghahramani, Z.: Dropout as a bayesian approximation: Representing model uncertainty in deep learning. In: International Conference on Machine Learning, pp. 1050–1059 (2016). Proceedings of Machine Learning Research
